# A Genetic Signature of Spina Bifida Risk from Pathway-Informed Comprehensive Gene-Variant Analysis

**DOI:** 10.1371/journal.pone.0028408

**Published:** 2011-11-30

**Authors:** Nicholas J. Marini, Thomas J. Hoffmann, Edward J. Lammer, Jill Hardin, Katherine Lazaruk, Jason B. Stein, Dennis A. Gilbert, Crystal Wright, Anna Lipzen, Len A. Pennacchio, Suzan L. Carmichael, John S. Witte, Gary M. Shaw, Jasper Rine

**Affiliations:** 1 Department of Molecular and Cellular Biology, California Institute for Quantitative Biosciences, University of California, Berkeley, California, United States of America; 2 Department of Epidemiology and Biostatistics and Institute of Human Genetics, University of California San Francisco, San Francisco, California, United States of America; 3 Children's Hospital Oakland Research Institute, Oakland, California, United States of America; 4 VitaPath Genetics, Inc., Foster City, California, United States of America; 5 Department of Energy, Joint Genome Institute, Walnut Creek, California, United States of America; 6 Department of Pediatrics, Stanford University School of Medicine, Stanford, California, United States of America; University of Bonn, Institut of Experimental Hematology and Transfusion Medicine, Germany

## Abstract

Despite compelling epidemiological evidence that folic acid supplements reduce the frequency of neural tube defects (NTDs) in newborns, common variant association studies with folate metabolism genes have failed to explain the majority of NTD risk. The contribution of rare alleles as well as genetic interactions within the folate pathway have not been extensively studied in the context of NTDs. Thus, we sequenced the exons in 31 folate-related genes in a 480-member NTD case-control population to identify the full spectrum of allelic variation and determine whether rare alleles or obvious genetic interactions within this pathway affect NTD risk. We constructed a pathway model, predetermined independent of the data, which grouped genes into coherent sets reflecting the distinct metabolic compartments in the folate/one-carbon pathway (purine synthesis, pyrimidine synthesis, and homocysteine recycling to methionine). By integrating multiple variants based on these groupings, we uncovered two provocative, complex genetic risk signatures. Interestingly, these signatures differed by race/ethnicity: a Hispanic risk profile pointed to alterations in purine biosynthesis, whereas that in non-Hispanic whites implicated homocysteine metabolism. In contrast, parallel analyses that focused on individual alleles, or individual genes, as the units by which to assign risk revealed no compelling associations. These results suggest that the ability to layer pathway relationships onto clinical variant data can be uniquely informative for identifying genetic risk as well as for generating mechanistic hypotheses. Furthermore, the identification of ethnic-specific risk signatures for spina bifida resonated with epidemiological data suggesting that the underlying pathogenesis may differ between Hispanic and non-Hispanic groups.

## Introduction

Neural tube defects (NTDs) are common, costly, and deadly human congenital anomalies whose causes remain largely unknown. The birth prevalence of NTDs varies from approximately 0.8/1,000 births in most areas of the US to 3.5/1,000 in Mexico [1]. Anencephaly and spina bifida are the most common forms of NTDs and result from failure of the neural tube to close properly in the developing brain or lower spine, respectively. Infants with anencephaly are stillborn or die shortly after birth, whereas many infants with spina bifida survive, but typically have severe, life-long disabilities.

Over 20 years of clinical investigation and studies with mouse NTD models indicate that these disorders arise from a combination of factors including complex genetic and gene-environment interactions [2], [3]. The most promising clue to the etiologies of NTDs, however, is that women who use vitamins containing folic acid periconceptionally (prior to and early in pregnancy) are at reduced risk for NTD-affected pregnancies [4], [5]. In addition, maternal use of anticonvulsants or other folic acid antagonist medications increases the occurrence of NTDs in offspring [6]. Taken together, these observations suggest that folic acid supplementation prevents NTDs by compensating for susceptibilities in folate transport, metabolism, or utilization. However, the underlying mechanisms by which folic acid contributes to these reduced risks are still unknown. Also unknown is why some women who take folic acid supplements in the periconceptional period still have offspring with NTDs.

The folate metabolic pathway plays critical roles in processes ranging from nucleotide biosynthesis, needed for cell proliferation, to generation of pterin cofactors impacting biochemical reactions, to generation of the principal methyl donor, S-adenosyl methionine (AdoMet), needed for methylation of DNA, proteins and lipids [7], [8]. Alterations in any of these processes may lead to folate-related pathologies. For example, decreased thymidylate synthesis results in increased uracil misincorporation into DNA and genomic instability [9]. Decreased AdoMet synthesis alters DNA and histone methylation, which can affect gene expression [7].

Because of this, multiple studies have explored possible associations between common single nucleotide polymorphisms (SNPs) in folate pathway genes and risk of NTDs [10]. Many of the known pathway SNPs have been evaluated, yet the results have shown either no or little association and many of the associations have not been consistently observed across studies. For example, as of 2009 there were 32 published studies of the association between the common 677C→T (A222V) variant of *MTHFR* and NTDs across many populations [1], [10]; half of these studies concluded that the 677T allele increased risk (usually when homozygous) whereas half found no statistically significant associations. A recent meta-analysis found association only in non-Latin populations, principally the Irish [11].

Other approaches have focused on identifying mouse genes that, when mutated, result in NTDs, hoping that human orthologs of such genes would be good candidates to harbor mutations that contribute to human NTDs. The potential complexity of NTD genetics is underscored by the more than 150 mouse genes implicated in NTDs which, for the most part, do not overlap with the folate metabolic pathway [3], [12]. Instead, these genes are centered around signaling pathways in development (such as non-canonical *WNT*), involved in cell morphology and differentiation [3], [12], [13]. Many of the mouse NTD models do not respond to folic acid supplementation [3], so it is unclear how well these models mimic human NTDs. Moreover, mouse studies tend to focus on null alleles, which could result in early prenatal lethality in humans. In any event, human homologs of some mouse NTD genes have been examined in association studies or directly sequenced in mutation screens, with few significant findings to date (with the possible exception of a functionally impaired mutation in VANGL1 in one NTD patient;[10]). However, a recent study with mice harboring mutations in the folate-related gene SHMT1 offer a breakthrough, establishing a folate-remedial NTD phenotype that interacts with NTD-disposing mutations in Pax3 *(Pax3^sp^*), a transcription factor involved in cell differentiation [14].

Thus, the multitude of genetic studies indicates that identifying specific NTD risk alleles has proven far from straightforward. The inconsistent results between different cohorts and populations for many common SNPs indicate that few, if any, of these SNPs have a major effect. These studies are complicated by several factors including: 1) the intricate interplay and cross-regulation between components of folate metabolism, 2) the potential number of genes participating in neurulation, and 3) the potential heterogeneity of the underlying mutation spectrum. To better unravel NTD genetics, it may be essential to evaluate multiple genes in the same individual to detect possible synergistic effects of combinations of risk alleles that, individually, would not be statistically or biologically significant [10]. For example, there are several examples in human [15] and mouse [16] suggesting that multiple genetic interactions underlie genetic susceptibilities that create NTD risk. Moreover, it may be important to consider gene variants in the context of metabolic pathway function, and how combinations of alleles impact pathway outputs. In addition, expanding consideration to rare or private mutations may be more effective than the historic focus on known, common polymorphisms as etiological determinants. Indeed, there is growing appreciation that common variants do not account for most of the heritability of many common diseases [17], [18].

This study used this more comprehensive SNP discovery and analysis approach. We sequenced the exons of 31 genes encoding enzymes central to folate metabolism in a population-based case-control study (N = 480). Our goal was to identify the full spectrum of allelic variation in folate pathway genes and determine whether rare alleles, combinations of alleles, or obvious genetic interactions within this pathway conferred NTD risk, specifically spina bifida. We found that analytical approaches that focused on individual alleles, or individual genes, as the units by which to assign risk did not show convincing disease associations. However, analyses based on simple pathway modeling that allowed us to infer metabolic consequences from groups of variants, and subsequently draw associations between the inferred metabolic impact and the NTD phenotype, revealed significant case-control differences. Furthermore, such “pathway level analysis” has indicated that the genetic contribution from folate pathway variation is both heterogeneous and mechanistically distinct in different races/ethnicities.

## Results

### Folate Pathway Sequencing in NTD Cases and Controls

We sequenced the coding regions of 31 genes in the folate-homocysteine metabolic pathway, comprising 430 coding exons, in 239 newborns with spina bifida and 241 non-malformed controls. We focused on coding regions because mutations in these regions are more likely to have functional impact, yet exhibit folate-remedial enzyme deficiencies [19], a characteristic that is consistent with and may underlie the folate-responsiveness of the NTD phenotype.

402 of the 430 target exons (>93%) gave robust sequence data with an average sample coverage of 90.6%. Of the 28 failed exons, 11 were from FOLH1, which has a nearby pseudogene. The number of variants by category for the 1,441 variants identified is summarized in Table 1. The exon resequencing strategy also produced a considerable amount of flanking non-coding sequence variation, which has proven quite informative (see below). The allelic variant calls were validated from a second, independent WGA of the bloodspot genomic DNA in two ways: 1) targeted re-sequencing confirmed over 90% of the original singleton calls (82 of 90 tested); 5 of the 8 that did not validate were either minor allele homozygotes or indels within homopolymeric tracts, and thus were already flagged; 2) Genotypes for 270 variants were determined in all 480 samples by TaqMan allelic discrimination assays and showed 99.8% concordance with the sequencing basecalls.

**Table 1:** Numbers of Variants Indentified by Category.

Variant Type	Number Identified	MAF≤2.5%	Singletons
**Non-Coding**	1050	776	406
**Nonsynonymous**	211	180	113
**Synonymous**	171	132	88
**Truncation**	4	4	3
**Frameshift**	5	4	4
**TOTAL**	**1441**	**1096**	**614**
**SNP**	1388	1055	585
**Insertion/Deletion**	53	41	29

### Individual Variants

The complete list of 1,441 variants is shown in Dataset S1 along with allele frequencies and case/control counts. We calculated univariate P-values for all variants seen multiple times (adjusted for race/ethnicity; see Methods). There were only 18 alleles that differed in frequency between cases and controls that had a P-value<0.05, and several of these were tightly linked to each other (Dataset S1). Only one of these 18 SNPs had been previously tested in another study (ATIC_14329/rs2372536;[20]) and in that study no association was found. Although these SNPs have suggestive P-values, it should be noted that because of the relatively large number of variants interrogated, even a liberal multiple comparison correction would have pushed all SNPs below a cutoff deemed significant. Thus, until these data are replicated, it is difficult to draw conclusions from such equivocal associations of individual variants. However, in this study we aimed to move beyond traditional univariate analysis, which has proven inconsistent in NTD genetics, by evaluating the potential role of the complete ensemble of gene variants in the folate pathway. Specifically, we investigated in parallel whether the aggregate burden of variants (particularly rare alleles) was associated with the NTD phenotype and whether there was evidence for folate pathway component interactions.

### Minor Allele Summing: All Genes/Individual Genes

We speculated that folate/one-carbon pathway function might be particularly susceptible to the aggregate mutation burden throughout the pathway, since there are many co-dependent and interacting components. In this way, the contribution of any one risk allele (or possibly even one gene) to pathway function might not be very significant in isolation, and thus explain the lack of heritability found in many common SNP-NTD association studies [10]. This limitation might be especially true for rare variants which are difficult to statistically analyze individually, but may be relevant when considered in aggregate [17], [21].

**Figure 1:**
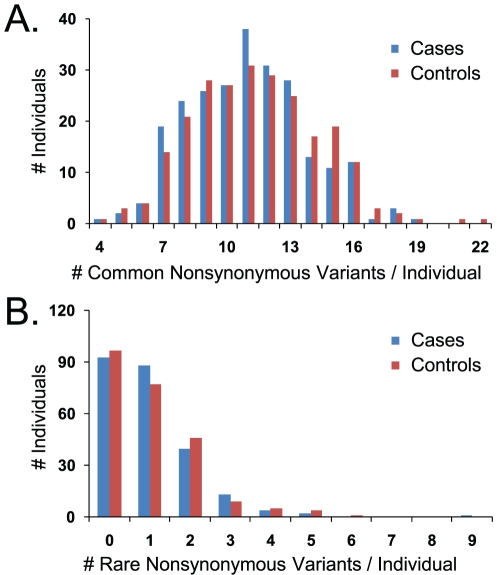
Total Nonsynonymous Changes in NTD Cases and Controls. A. All common nonsynonymous alleles (MAF>2.5%) identified from the 31 folate pathway genes sequenced were summed in each individual and the case/control distributions of these sums are shown. Minor allele homozygotes were counted as two alleles whereas heterozygotes were counted as one. B. Case/control distributions of rare (MAF≤2.5%) nonsynonymous allele sums. Population means and permuted P-values are shown in Table 2.

We performed various minor allele summing/collapsing analyses in which we simply tallied the number of minor alleles (counting 1 for a heterozygote and 2 for a homozygote) in the 31 sequenced genes as a straightforward measure of mutation burden in the pathway. However, such analysis for nonsynonymous SNPs did not reveal statistically significant differences between cases and controls (Figure 1). This lack of a signal was true whether we considered only common variants (defined here as MAF ≥ 2.5%; Figure 1A), or only rare variants (MAF<2.5%; Figure 1B). The significance levels (permuted P-values) for these and other case-control comparisons using different variant subsets are described in Table 2. Also shown are comparisons for the two most frequent race/ethnicities: Hispanics and non-Hispanic whites (65% and 21% of the samples, respectively). We found it more informative to perform these analyses following race-ethnic stratification which enabled us to query whether any NTD-associated genetic signatures were common to both groups. All comparisons failed to reveal a statistically significant difference (permuted P-value≤0.05, not adjusted for multiple comparisons) irrespective of the variant subset considered (including variants not shown such as synonymous changes). Testing these distributions via Kolmogorov-Smirnov goodness-of-fit (rather than means) did not change these results [22]. Thus, the hypothesis that a cumulative mutation burden across the entire pathway was responsible for NTD risk was not supported by the data when all genes were aggregated and weighted equally (Table 2).

**Table 2:** Mean Number of Minor Alleles from 31 Folate Pathway Genes in Cases vs. Controls for Different Variant Subsets.

Variant Subset	All Cases	All Controls	Permuted P-Value	Hispanic Cases	Hispanic Controls	Permuted P-value	White Cases	White Controls	Permuted P-value
COMMON a									
All	116.8±13.2	119.1±15.3	**0.1**	115.3±12.9	117.7±14.1	**0.12**	125.7±10.8	125.5±15.4	**0.95**
Nonsynonymous	11±2.8	11.3±3	**0.2**	11±2.6	11.5±3	**0.12**	12.2±3.2	12.5±2.9	**0.65**
Non-Coding	91.2±11	93.4±13.2	**0.06**	90.1±10.5	92.3±12.1	**0.13**	95.8±10.2	97±12.9	**0.62**
RARE a									
All	7.8 (1–54)	7.8 (0–42)	**0.54**	6.1 (0–20)	6.1 (0–22)	**0.82**	5 (0–34)	4.4 (0–13)	**0.73**
Nonsynonymous	1 (0–9)	1 (0–6)	**0.96**	0.9 (0–5)	0.9 (0–5)	**0.93**	0.7 (0–4)	0.5 (0–2)	**0.6**
Non-Coding	6.2 (0–43)	6.2 (0–38)	**0.47**	4.7 (0–18)	4.7 (0–19)	**0.81**	3.8 (0–29)	3.5 (0–11)	**0.48**

a Common alleles are defined as MAF≥2.5% within the specific race-ethnic group analyzed, whereas rare alleles are MAF<2.5%. Means are shown±standard deviation for common variant analyses where the distributions were near normal. For rare variants, the distributions were tailed (see Figure 1B) and the range of values is provided in parentheses. Permuted P-values were calculated as in Materials and Methods, and were not adjusted for multiple comparisons.

**Figure 2:**
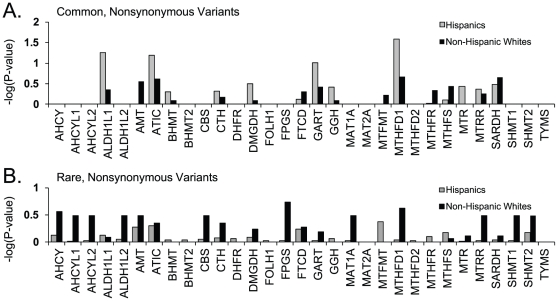
Allele Sums by Individual Gene. Common and rare nonsynonymous changes were summed as in Figure 1 except here on a gene-by-gene basis rather than considering the pathway as a whole. –log (P-values) were calculated by permutation (but not adjusted for multiple comparisons) based on the case/control distributions within each gene in Hispanics and non-Hispanic whites. On the y-axis, 1.3 corresponds to P-value = 0.05.

To determine whether there might exist subsets of this 31-gene set that may harbor more significant case-control differences, we then used these same variant collapsing approaches to look at the contribution of individual genes, rather than the entire group, to the NTD phenotype. Again, the collection of common and/or rare nonsynonymous SNPs similarly analyzed in each gene did not convincingly distinguish cases from controls for most genes (Figure 2). The most suggestive result came from collapsing common nonsynonymous SNPs in *MTHFD1* in the Hispanic sub-population (permuted P-value = 0.04, not adjusted for multiple comparisons). Interestingly, a common nonsynonymous SNP in *MTHFD1* (R653Q) has been previously identified as a NTD risk factor in Irish and Italian populations [23], [24]. Changing the MAF cut-off used to differentiate common and rare alleles to 1% or 5% did not materially change the results (data not shown), nor did the use of more complex analytical methods that weight variants [25], [26] or group variants by statistical criteria [27]. Furthermore, the case-control distributions for other variant subsets (e.g. non-coding) displayed no significant differences for individual genes. Thus, this gene-level analysis did not strongly indict any of these genes in NTD risk.

### Minor Allele Summing: Pathway Model

Many proteins play important roles in folate metabolism and the pathway is characterized by multiple interconnected cycles with multiple metabolic branch points and feedback regulatory mechanisms [28]. At the outset of the study, we therefore reasoned that simply measuring the pathway-wide accumulation of mutations, or considering genes or alleles in isolation, might not adequately take into account this interplay. Indeed, mutations in certain genes might be expected to be deleterious to one set of reactions, but advantageous to others, because that enzyme competes for substrates with other enzymes, or drives a reaction in an opposite direction. If the NTD phenotype were affected by pathway function, then a more relevant measure for disease risk would be metabolic output, which would be due to the integrated effect of numerous variants across the pathway.

To this end, we developed a simple model to investigate the integrated effect of multiple genetic changes on three key metabolites relevant to folate metabolism: thymidylate synthesis, purine synthesis, and homocysteine recycling. In this model, we incorporated mostly genetic and cell-based observational studies from the literature to derive relationships between enzymes and their effects on pathway flux for the various metabolites. With this information, we identified subsets of the pathway relevant to each metabolite and further inferred whether gene function would contribute positively or negatively to that process. We then signed each variant allele in each gene accordingly, with a “+” or “−”, to reflect its potential impact. In this method, for example, minor allele homozygotes in a gene whose function is beneficial to a process are assigned +2, whereas heterozygotes in a gene whose function may compete with the process are assigned −1. The resulting sum of alleles in a particular metabolic compartment was a single number (referred to as the Metabolic Index Score or MIS) that reflected the mutational load on that process in that individual. It should be emphasized that these designations were all made prior to data analysis and, thus, were not biased by any observed trends in the variant data. Analysis based on pathway relationships was carried out independently and in parallel with the allele- and gene-level analyses described above.

This was a relatively simple model because folate levels were not explicitly accounted for. In addition, each gene (whether positive or negative), and each mutation within a gene, were given identical weight. This strategy was in contrast with previously described, more mathematically-intensive models that take into account enzyme-specific parameters and metabolite levels [28]–[30]. Nevertheless, there was a good deal of qualitative agreement between these two methods of designation.

**Figure 3:**
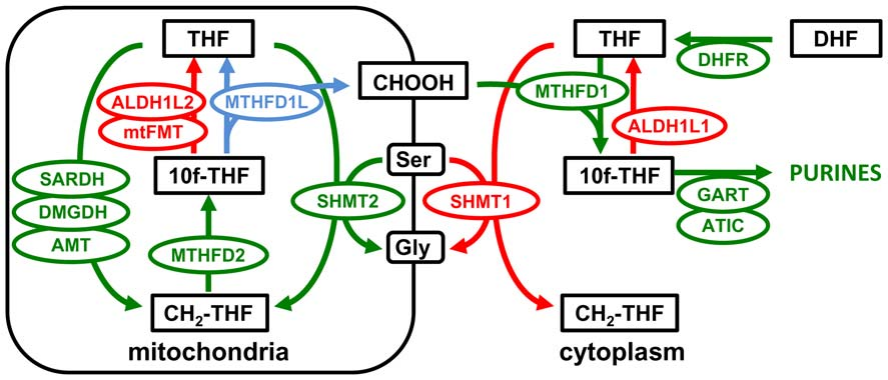
Purine Metabolic Cassette. The subset of reactions within the folate metabolic pathway that have been inferred to be relevant to purine biosynthesis based on observations in the literature (see text for details). Genes (balloons) and their cognate enzymatic steps (arrows) are colored either green (beneficial to purine synthesis) or red (detrimental to purine synthesis). The MTHFD1L reaction, responsible for generating mitochondrial formate [31] and beneficial to purine synthesis, is shown in blue because it was not one of the 31 sequenced genes.

As an example, the subset of folate pathway genes that we defined as particularly relevant for purine synthesis and their inferred relationships governing metabolite flow for this process are illustrated in Figure 3. Our inferences were based on the following considerations. In general, the conversion between tetrahydrofolate (THF) and 5,10 methylene-THF (CH_2_-THF) operates in the oxidative direction in the mitochondria (CH2-THF → THF via MTHFD2 and MTHFD1L reactions), but in the reductive direction in the cytoplasm via MTHFD1 [31], [32]. Though MTHFD1 is a tri-functional enzyme in this conversion, only the formyl-THF synthase activity is shown in Figure 3, which utilizes mitochondrially-produced formate (CHOOH) in the synthesis of 10-formyl-THF (10f-THF) in the cytoplasm. This intermediate is an essential carbon donor at two distinct steps in purine biosynthesis (catalyzed by ATIC and GART), thus *MTHFD1* inactivation is lethal in mice and results in purine auxotrophy in cultured cells [9], [33].

Under most conditions, the majority of one-carbon units for cytoplasmic production of purines and methionine are derived from mitochondrial formate, which is produced by the formyl-THF-dehydrogenase MTHFD1L [9], [31]. Thus all reactions that feed into the MTHFD1L reaction (MTHFD2, SHMT2, AMT, SARDH, DMGDH) were considered beneficial for purine synthesis, whereas reactions that may compete with MTHFD1L in formate generation (MtFMT, ALDH1L2) were considered deleterious.

Similarly, cytoplasmic ALDH1L1 was considered deleterious to purine synthesis because it would compete with GART/ATIC for 10f-THF. Although high levels of ALDH1L1 do not appear to specifically deplete 10f-THF, there is a general depletion of cellular folates (especially 5-methyl-THF) indicating perturbations to flux in the reductive direction [34]. SHMT1 has been designated as negatively affecting flux to purines because mice with decreased MTHFD1 activity show enhanced de novo thymidylate synthesis, suggesting that SHMT1 and MTHFD1 compete for a limiting pool of THF in the cytoplasm [9].

We have also designated coherent, signed gene sets for thymidylate synthesis and homocysteine levels (Table S3) in a similar way. Homocysteine-related genes overlap the purine group (but contain more genes) because they were designated by much of the same reasoning. For example, several studies link mitochondrial formate to the remethylation of homocysteine as it enters the methyl cycle [9], [31], [35]. The additional genes in the homocysteine group are mostly methionine cycle genes (BHMT,BHMT2,MTHFR,MTR,MTRR) and trans-sulfuration pathway genes (CBS,CTH) whose collective activity can drive homocysteine utilization into either of these processes (and thus negatively impact homocysteine levels). MAT1A and MAT2A (which synthesize AdoMet directly from methionine) are excluded from the homocysteine gene set since AdoMet levels do not have a major effect on homocysteine levels, though AdoMet can influence whether homocysteine is metabolized via the methionine cycle or trans-sulfuration (through allosteric inhibition of MTHFR and activation of CBS;[36]). For most genes present in both the purine and homocysteine gene sets, the signing to derive the Metabolic Index Score is opposite because a reduced pathway flux would have a negative effect on purine synthesis, but a positive effect on homocysteine levels (less homocysteine would be utilized for methionine/cysteine synthesis).

**Figure 4:**
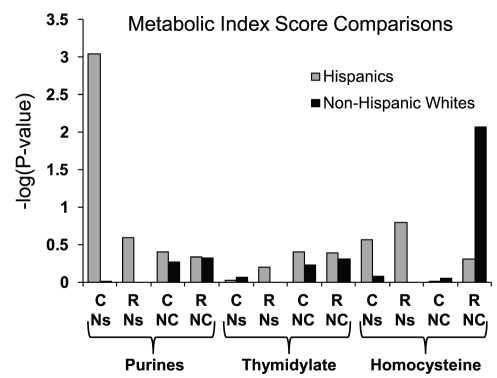
Inferred Metabolic Impact in NTD Cases and Controls. Metabolic Index Scores were calculated for 3 key folate-regulated metabolites (Purines, Thymidylate, and Homocysteine) and differences in the case/control distributions are represented by –log(P-values). Index Scores were derived from alleles summed according to pathway designations (gene sets and directionality; see Figure 3 and Table S3) and P-values following permutation were calculated as described in Methods. The P-values shown are not adjusted for multiple comparison testing. For each metabolite, 4 different subsets of variants were considered to derive Index Scores and are indicated by the following abbreviations on the x-axis: C, common alleles (MAF>2.5%); R, rare alleles (MAF≤2.5%); Ns, nonsynonymous variants; NC, non-coding variants. Results are shown for Hispanic and non-Hispanic white subsets of the study population.

For thymidylate, our relevant gene set was centered around the module consisting of SHMT1-TYMS-DHFR because of several lines of compelling evidence: First, these 3 enzymes exhibit regulated translocation from the cytoplasm into the nucleus during S-phase to compartmentalize dTMP synthesis [37], [38]. Second, SHMT1 loss results in decreased thymidylate synthesis [39], whereas MTHFD1 defects may increase thymidylate synthesis by allowing more THF into the SHMT1 reaction [9]. In addition, we factored in the observation that MTHFR can compete with TYMS for CH_2_-THF and thus have a negative impact on dTMP synthesis [35], whereas the downstream enzymes in the methionine cycle responsible for AdoMet production (MTR,MTRR,MAT1A,MAT2A) might have a positive effect on dTMP synthesis due to the AdoMet-mediated inhibition of MTHFR. This integrated approach, which accounts for relevant pathway interactions, revealed statistically significant NTD-associated signals following stratification of the study subjects by race/ethnicity (Figure 4). For each pathway compartment, we drew case-control comparisons of the MIS using 4 different variant subsets, signed and summed as described above. For the Hispanic sub-population, a strong discriminatory signal emanated from common, nonsynonymous SNPs (15 variants; unadjusted P-value = 0.0009) in genes binned as relevant to purine synthesis. For non-Hispanic whites, this signal was absent, but a suggestive signal unique to this group (unadjusted P-value = .008) was derived from the collection of rare, non-coding variants (n = 195) in homocysteine-related genes. Adjusting the P-values for multiple comparisons (most of which are shown in Table 2 and Figure 4) by max(T) permutation [40] resulted in a P-value of 0.024 for the purine-related signal in Hispanics and a P-value of 0.13 for the homocysteine-related signal in non-Hispanic whites. These results suggested that these metabolic processes may be altered in NTD cases and, intriguingly, that the underlying mechanisms of NTD risk may be different in these two groups.

### Hispanic Signature in Purine-Related Genes

**Figure 5:**
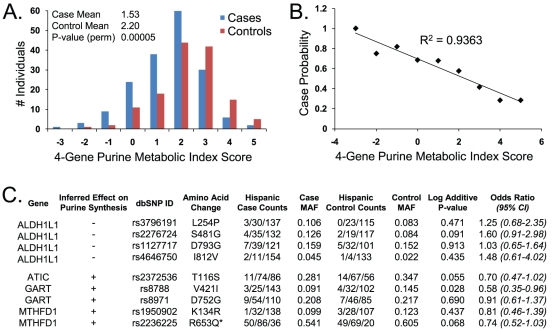
Nonsynonymous Variants in the Hispanic Signature. A. Hispanic case/control distributions of purine Metabolic Index Scores calculated from common synonymous variants (n = 9) in 4 genes (*ALDH1L1, ATIC, GART, MTHFD1*). The P-value shown is not adjusted for multiple comparisons (see text). B. The data in panel A transformed to demonstrate the fraction of cases as a function of purine Metabolic Index Score. C. Attributes of the 9 SNPs that went into the purine MIS calculations of panel A. “Inferred Effect on Purine Synthesis” is the inferred effect of that particular gene product on purine biosynthesis (see text for details). Case and control counts are given as individual genotype distributions: # minor allele homozygotes/# heterozygotes/# major allele homozygotes). Log additive P-values and odds ratios were calculated as in Methods using only Hispanic samples and not multiple-testing adjusted. *Minor Q653 MTHFD1 enzyme variant is the major allele in Mexican-American populations (http://hapmap.ncbi.nlm.nih.gov/).

The folate-mediated enzymatic steps necessary for purine synthesis occur in both the cytoplasm and mitochondria (Figure 3). To determine if the metabolic contribution from both compartments was represented in the Hispanic signature, we subsequently analyzed the subsets of purine-related genes specific to each compartment. This analysis clearly indicated that the signal principally derived from 4 genes encoding cytoplasmic enzymes: *ALDH1L1, MTHFD1, ATIC*, and *GART* (Figure 5). These genes contained a total of 9 common, nonsynonymous SNPs. Interestingly, some of this signal was also hinted at from individual gene analysis (Figure 2), since these 4 genes displayed the greatest case-control differences in Hispanics, though only *MTHFD1* exhibited a P-value<0.05. Significantly, these 4 gene products converge on cytoplasmic 10f-THF, a metabolite critical for purine synthesis.

Comparing those high in the purine MIS derived from these 9 variants to those low in the score was highly associated with case status (P-value = 0.00005; P-value = 0.001 when adjusted for multiple comparisons; Figure 5A). Indeed, there was a strong correlation between the MIS and the probability of case status (Figure 5B). The odds ratio based on the Hispanic control population median (MIS = 2) was 0.26 (95% CI 0.15 – 0.47), whereas an odds ratio calculated on only the 17% of the population at the extremes of the distributions (Metabolic Index Scores≤−1 or ≥ 4; n = 44) was 0.09 (95% CI 0.02 – 0.41). This is an unusually large magnitude of effect compared to previous reports for NTD association studies. The odds ratios are less than 1 because this risk profile is one where the mutation load, as evidenced by the Metabolic Index Score, confers a reduced risk of case status. The implications of a lower MIS in cases is discussed below.

The 9 SNPs in this signature and their attributes are listed in Figure 5C. It is worth noting that the combination of genotypes from this group was very heterogeneous in the population. For example, in 173 Hispanic cases, there were 110 distinct genotype combinations at these 9 sites. Furthermore, only one of these 9 SNPs was individually mildly suggestive with respect to NTD association in this Hispanic population (GART V42I, P-value = 0.028; Figure 5C). Thus, it appeared that rationally integrating this set of variants in a biological context was required to reveal a statistically significant trend. For example, note that all nonsynonymous variants in ALDH1L1, which was designated as deleterious to purine synthesis (signed “-”), were over-represented in cases (ORs > 1), whereas the variants in genes designated as beneficial to purine synthesis were over-represented in controls (ORs<1). The allele frequencies for these variants in the Hispanic controls were in good agreement with HapMap 3 data for individuals with Mexican ancestry. Thus our population was not skewed in this respect from the frequencies expected from this community (http://hapmap.ncbi.nlm.nih.gov/).

Though these associations have not yet been formally validated in a second population, the signal is quite strong in Hispanics and was present in all subsets of the Hispanic population tested. For example, case-control differences in the 4-gene purine MIS were significant in children from Hispanic mothers born outside the United States (n = 224; unadjusted P-value = 0.0005) and suggestive in children from U.S.–born Hispanics (n = 87; unadjusted P-value = 0.01). In addition, significant differences were seen in Hispanic samples collected from 1984 to 1986, prior to mandatory folic acid fortification of grain products ([41]; n = 183; unadjusted P-value = 0.002) as well as in Hispanic samples collected post-fortification (1999-2003; n = 128; unadjusted P-value = 0.009).

### Non-Hispanic White Signature in Homocysteine-Related Genes

**Figure 6:**
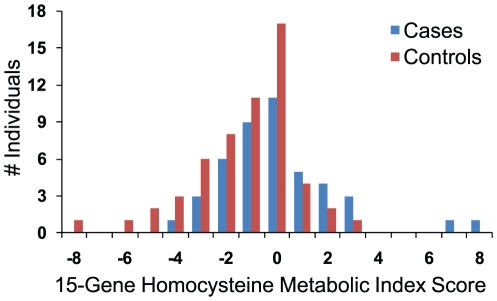
Homocysteine-Related Signature in Non-Hispanic Whites. Non-Hispanic white case/control distributions of homocysteine Metabolic Index Scores calculated from rare, non-coding variants (n = 149) in 15 homocysteine-related genes, signed and summed according to Table S3. See text for details.

From our pathway-level analysis, a homocysteine metabolism genetic signature that significantly distinguished cases from controls was observed only in the non-Hispanic, white population (Figure 4), suggesting that homocysteine recycling may be implicated in NTD risk for this group, a link that has been drawn previously [42], [43]. Unlike the purine synthesis signature, this homocysteine signal emanated from a large number of rare, non-coding variants signed and summed as described above. Upon further dissection of this signal, we found that, similar to the purine signature above, genes encoding mitochondrial enzymes did not appreciably contribute to the case-control differences. A Metabolic Index Score comparison built only on the 15 cytoplasmic genes (which harbor 149 rare, non-coding variants) designated as relevant to homocysteine metabolism (see Table S3) is shown in Figure 6. The signal was suggestive of an association (P-value = 0.004; P-value = 0.076 when adjusted for multiple comparisons) and was the most noteworthy trend seen in the non-Hispanic white sub-population. The odds ratio comparing individuals with MIS below 0 to those above was 3.4 (95% CI 1.2 – 9.8).

## Discussion

This report described a large and comprehensive variant discovery effort that identified the full spectrum of allelic variation in nearly the entire folate metabolic pathway in spina bifida cases and non-malformed controls to evaluate the contributions of rare variants and pathway interactions to disease risk. We interpreted the resulting variant data at several levels from traditional single-allele associations, to rare-variant summing methods, to defining relevant genetic interactions based on folate pathway relationships to infer metabolic consequences. As described above, all analyses were performed independently in parallel following completion of the variant dataset.

Analytical approaches that focused on individual alleles, or individual genes, as the units by which to assign risk did not show convincing disease associations. However, analyses that accounted for pathway function through a simple model revealed two provocative, complex genetic signatures that showed compelling statistical evidence for distinguishing cases from controls. In one, a genetic signature that strikingly differentiated between Hispanic cases and controls derived from the combined effect of folate pathway genes related to purine biosynthesis, whereas a risk signature in non-Hispanic whites emanated from genes related to homocysteine metabolism.

These genetic risk profiles appeared to be ethnically specific, indicating the underlying disease mechanisms may also be different. These findings, which emphasized genetic heterogeneity and interactions among multiple genes, may explain why results from previous association studies have been inconsistent. In addition, these findings may provide some mechanistic insights into the well-described (but poorly understood) differences in NTD links with nutritional status, genetic susceptibilities and Hispanic ethnicity.

### Purine Synthesis and NTDs

Evidence implicating purine synthesis in NTD risk has come from the repeated identification of a common, nonsynonymous risk allele in *MTHFD1* (1958 G→A; R653Q) [23], [24]. This amino acid substitution mildly impairs enzyme thermostability in vitro, but markedly impairs de novo purine synthesis in cells [44]. This variant was not an independent risk factor in our study, but was part of the Hispanic purine signature. Interestingly, the Q variant is the minor allele in all race-ethnic groups tested except those of Mexican ancestry (http://hapmap.ncbi.nlm.nih.gov/; Figure 5C), in which the NTD risk is significantly greater than in non-Hispanic whites or African Americans [45], [46]. Furthermore, in one mouse model of NTDs, homozygous *Splotch* mutant (*Pax3^sp/sp^*) embryos exhibit fully penetrant, yet folate-remedial, spina bifida [47]. Metabolic experiments with embryonic fibroblasts from *Pax3* mutant mice suggest that the major defect is de novo purine synthesis [14].

In developing embryos, the concerted efforts of MTHFD2 and MTHFD1L (to generate formate in the mitochondria) and MTHFD1 (to incorporate formate into 10f-THF) are essential for purine synthesis [31], [32]. This dependence on de novo purine synthesis is apparently restricted to developing embryos, since adult mitochondria lack MTHFD2 and MTHFD1L activity and produce significantly less formate than embryonic mitochondria [31]. Interestingly, MTHFD2, MTHFD1L and MTHFD1 share a similar spatial pattern of expression in developing mouse embryos with the highest levels of expression in the developing brain, craniofacial structures, limbs/digits, neural tube, and tail bud, which are all undergoing high levels of cell division [31]. While no variants in *MTHFD2* have been associated with NTD risk, a common, short-repeat variant in *MTHFD1L* that affects mRNA splicing has been associated with risk in an Irish population [48].

### Homocysteine Metabolism and NTDs

A homocysteine metabolism genetic signature that significantly distinguished cases from controls was observed only in non-Hispanic whites (Figures 4,6]. This risk signature emanated from variants in non-coding regions, suggesting that effects on gene expression underlie this signal. This finding resonates with earlier data in which homocysteine was already implicated as a risk factor for NTDs [42], [43], presumably due to the relationship between elevated homocysteine levels and perturbations of the methylation cycle [49]–[52]. Furthermore, this signature was found in the racial group for which the strongest evidence of its contribution to risk exists (see above). It should be noted, however, that in most association studies homocysteine levels are typically assessed in mothers after delivery, whereas the genetic signature described here is fetal. Whether this is a function of transmission of maternal genetic defects (in which case the signature would presumably be stronger in mothers) or indicates fetal metabolic deficiencies is unknown. In either case, this complex signature was identified from a relatively small white population (44 cases/56 controls) and, although statistically meaningful, needs to be replicated in a larger population.

It should be emphasized that we surveyed only the non-coding regions that were immediately adjacent to the target exons of these genes and, therefore, represented only a small fraction of the non-coding DNA within these genes. Whether we increased our chances for finding meaningful variants by focusing on regions close to exons is unknown, but this finding warrants further exploration into non-coding DNA. Non-coding mutations have also been implicated in murine models of NTDs, such as the ct/ct mouse [16].

### The Metabolic Index Score

The MIS was a simple metric designed to incorporate biologically relevant interactions among alleles to infer a physiological outcome. Relationships were designated based mostly on phenotypes from genetic and cell-based studies and thus it is a somewhat qualitative tool. Nevertheless, there was good agreement between relevant gene sets defined here and those from a more mathematical model of folate metabolism that is based on intracellular folate concentrations, enzymatic kinetics, allosteric inhibition, and known polymorphism-biomarker relationships [28]–[30].

One aspect of the Metabolic Index Scores reported here merits further discussion. Since the MIS is indicative of the mutational burden expected to have a negative impact on a given process, it was somewhat surprising that the Hispanic cases are set apart from controls in the purine synthesis signature because cases have a *lower* average score. Thus, the inferred metabolic impact would be that of increased purine synthesis in cases relative to controls. A similarly surprising inference was drawn from the homocysteine risk signature in non-Hispanic whites. In this population, cases have a higher average MIS (reflecting the mutational burden on homocysteine abundance; Figure 6), suggesting a potentially lower level of homocysteine than in controls. However, one might have expected the opposite, given the previously observed relationship between elevated homocysteine and NTD risk (see above). Thus, in both instances, the MIS suggested an unexpected metabolic consequence.

Metabolic inferences from these observations should be drawn with caution for several reasons: In a pathway with many feedback regulatory mechanisms, response to certain perturbations can have unexpected consequences. In other words, a reduction (or gain) of activity at certain pathway reactions may result in a metabolic profile that was not predicted by that change. For example, overexpression of ALDH1L1 (which converts 10f-THF to THF by deformylation) results in a higher ratio of 10f-THF:THF when the opposite would be expected [34]. Furthermore, we do not yet know the functional impact for most variants and which, if any, alleles may be gain-of-function variants. Although, the MTHFD1 R653Q variant has a slight thermostability defect [44], other missense changes in the purine set have not been empirically tested. The functional impact of non-coding changes is more difficult to assess. Moreover, we do not yet know whether alleles may exhibit stronger phenotypes in combination. Because of these caveats, there may not be a straightforward relationship between a higher Metabolic Index Score and reduced metabolic flux.

Alternatively, there are examples in which major alleles can be risk alleles, while minor alleles may be protective. For example, in individuals with nonsyndromic orofacial clefts, the major allele for common polymorphisms in *IRF6* and *FOXE1* are over-represented [53]–[55]. Likewise in *MTHFD1L*, the major non-coding allele in Northern European populations (which is thought to retain correct mRNA splicing) may increase risk for NTDs in the Irish, whereas a minor defective allele may reduce risk [48]. These observations may indicate that some metabolic “inefficiencies” are beneficial. For example, purine synthesis may compete with homocysteine remethylation for one-carbon units [31], [34]. Therefore, an increase in flux into the purine compartment (which may be inferred from the lower MIS in Hispanic cases) may compromise homocysteine remethylation and, consequently DNA methylation.

Ultimately, the mechanisms behind this or any disease-risk genetic signature will have to be addressed empirically by direct metabolic measurements rather than relying solely on inferences and models. The approach described here, whereby biological principles are employed to focus on relevant allelic interactions, can catalyze specific tests of the impact of risk signatures on measurable metabolites and, thus, provides multiple avenues for supporting or rejecting mechanistic hypotheses.

### Is it Surprising that NTD Genetics may Differ between Hispanics and non-Hispanics?

A race-ethnic difference in underlying NTD genetics would be consistent with several epidemiological studies. For example, folate supplementation confers less risk reduction among Hispanic (primarily Mexican) populations [56], [57]. Because Mexican-Americans have 2 – 3 times higher risk for NTDs than those of non-Hispanic whites and African Americans [45], [46], these studies suggested that conventional levels of folate intake (via diet or supplements) may not adequately protect this population. We speculate that Mexican Americans may require higher intakes of folate to prevent NTDs to compensate for a unique signature of underlying susceptibilities in the folate pathway.

Furthermore, our observation that a homocysteine-relevant signal is seen in non-Hispanic whites, but not Hispanics, is consistent with studies surrounding the common 677 C→T (A222V) variant of *MTHFR* [10]. This variant has received considerable attention (with mixed results) because it results in an impaired enzyme that can be remediated by folate supplementation [58]. Furthermore, reductions in MTHFR activity result in accumulation of homocysteine and a subsequent perturbation of the methylation cycle [49], [50]. The most recent meta-analysis of this allele [11], did not find a positive association in Hispanic groups when stratified by ethnicity, and suggested that non-Hispanic descent could be a requirement for the association of NTDs and *MTHFR* 677C→T.

### Strengths/Weaknesses of Study

The strengths of this study are two-fold. First, we have completed a sizeable and unique effort to catalogue both common and rare variation in nearly the entire folate pathway as it relates to the NTD phenotype. Furthermore, we have presented a unique way in which to analyze allelic interactions by integrating the variant data in the context of a folate pathway model to infer metabolic outcomes from multiple alleles in a single individual. Such an exploratory genetic analysis revealed significant case-control differences in our population. One limitation in our analysis, however, is that the pathway relationships that we designated to guide whether variants may synergize or compensate involved two sets of assumptions, both of which were broadly reasonable, though in detail may not accurately reflect pathway function for every individual. First, we assigned subsets of the folate pathway as more relevant to certain metabolites based on observations in the literature. Almost certainly, all of the metabolic processes discussed will be affected by more genes than we considered (including those encoding proteins outside of folate-related enzymes). Thus, the extent to which our model re-created all relevant interactions accurately is unknown. In addition, to simplify analysis, all genes (and all variants within the genes) were equally weighted. Therefore all are assumed to have the same, negative impact on gene function and contribute equally overall to the particular process. However, we do not know the functional impact of most variants and thus it is possible that we are scoring benign alleles incorrectly. Formally it is also possible that a small subset of mutations could be activating rather than inactivating as some enzymes have regulatory domains. This issue may also be particularly relevant for the homocysteine signature because it emanated from non-coding alleles whose functions are difficult to infer. Indeed, it is unlikely that all of the rare alleles included to derive the homocysteine Metabolic Index Score have functional consequence. In fact, a knowledge of which alleles are functionally altered and which are benign may sharpen trends in these associations. Nevertheless, a successful implementation of this approach does not demand that all assumptions be correct, but rather that we have captured enough biological relevance to detect a signal above the noise. In this regard it will be imperative to replicate these findings in additional populations; particularly in non-Hispanic whites were our current sample size is small.

## Materials and Methods

### Sample Population

This case-control study included data on deliveries that had estimated due dates from 1984–86 or 1999–2003. The study included liveborn infants with spina bifida (cases; N = 241) or without any malformation (controls; N = 239) identified by the California Birth Defects Monitoring Program. At the time of collection, parents/guardians were given the opportunity to have any residual sample (remaining after newborn genetic screening) removed from future state-approved health research. Only individuals whose parents or guardians did not elect to have their samples removed from such future studies were included. The race-ethnic breakdown of the study samples is in Table S1. Case information was abstracted from hospital reports and medical records following established procedures by the California Birth Defects Monitoring Program [59]. Each medical record was further reviewed by a medical geneticist (E.J.L.). Infants with trisomies were ineligible. Controls were selected randomly to represent the population from which the cases were derived in selected counties and birth periods. This study, including the collection and use of archived newborn bloodspots, was approved by the California State Committee for the Protection of Human Subjects as well as Institutional Review Boards at Stanford University and the University of California, Berkeley.

### Sample Prep/Bloodspot workup

Genomic DNA from each individual was isolated from a single surgical bloodspot punch (2 mm dia.) using the QIAamp DNA Micro Kit (Qiagen) according to the manufacturer, with a final column elution volume of 25 ul. Average yield was 40 ng gDNA/punch. 5 ul of the gDNA prep was whole-genome amplified (Repli-G Midi, Qiagen) and subsequently purified on QIAamp DNA mini columns (Qiagen) according to the manufacturer. The average yield of purified whole-genome amplified (WGA) DNA was 15 ug per individual.

### DNA Sequencing

The coding regions of 31 folate-related genes (Table S2) were sequenced in each individual by PCR/sequencing using exon-specific primers as described previously [60]. Because primer design captured a substantial amount of non-coding DNA from adjacent introns with potential regulatory sites, variants in these regions were also catalogued and incorporated into analyses. Quality and accuracy of sequence data were evaluated by performing a second WGA on gDNA isolated from each punch and confirming, 1) a subset of common SNP genotypes with TaqMan allelic-discrimination assays, and 2) a subset of singletons by re-sequencing. See Results section for more details.

### Imputation of Missing data

Prior to all analyses, we discarded individuals and genotypes with more than 25% missing data. This excluded 3 individuals and 141 of 1441 total variants called. For the remaining 1300 variants, missing data (5.9%) were imputed via the program Beagle v3.0.4 [61].

### Analyses of Single Alleles

Associations between genotypes and spina bifida were assessed assuming an additive model of inheritance. Associations calculated from the entire study (those shown in Dataset S1) were adjusted for race/ethnicity. Odds ratios and p-values were generated using exact inference for logistic regression as implemented in the R package *elrm* [62]. All models were undertaken with R software, version 2.10.1.

### Allele Summing Analyses

As a measure of mutation burden, unweighted minor allele sums (1 for a heterozygote, 2 for a homozygote) were calculated in each individual for each gene (or group of genes) using various genetic subsets (e.g. rare, nonsynonymous mutations). Allele sums for common variants (Minor Allele Frequency (MAF) ≥ 2.5%), or common plus rare, were approximately normally distributed in case and control populations and differences in the means between these groups were evaluated using the student's T-test. For rare alleles (MAF<2.5%), the distributions were skewed toward fewer numbers of alleles and differences between case and control distributions were estimated by the Mann-Whitney U test. Final P-values were calculated by permuting the case-control labels.

### Pathway-based Analysis

Prior to any data analysis, we constructed a simple pathway model (see Results for details) in which individual genes were considered as either beneficial or deleterious to a particular pathway function (e.g. thymidylate synthesis). Then based on this, allele sums for a group of metabolically-related genes were obtained by adding the number of beneficial variants in some genes while subtracting the number of deleterious variants in other genes. These sums, from which we inferred metabolic impact on pathway function, were approximately normally distributed in cases and controls and, as above, were compared with the student's T-test permuting the case-control labels. In addition (where indicated in the text), all pathway combinations were corrected for multiple comparisons by the max(T) permutation procedure [40]. For all allele-summing analyses, variants that were in linkage disequilibrium with a R^2^ > 0.8 were represented by a single allele from that LD group.

## Supporting Information

Table S1 **The race-ethnic breakdown of the cases and controls in the study population.**
(DOC)

Table S2 **The list of folate-related genes (with NCBI identifiers) whose coding regions were sequenced.**
(DOC)

Table S3 **Pathway designations for thymidylate synthesis, homocysteine levels and purine synthesis.** Gene sets inferred to be relevant for a metabolite are indicated by a “+” or “–” sign, which indicates the inferred effect that gene product exerts on metabolite levels and, thus, whether variants in those genes are added to or subtracted from the Metabolic Index Score (see text for details). Those without a sign are not factored into the pathway-level Metabolic Index Score for that metabolite.
(DOC)

Dataset S1 **The list of 1441 variant positions identified in the study.** Variant ID is in the form “GENE_Locus Coordinate” where position #1 is -1000 nucleotides from the transcription start site according to the refseq IDs in Table S2. The first 18 variants were the only ones in the study to display P-values < 0.05 using a log-additive disease model, adjusted for race-ethnicity, and for which odds ratios were calculated. Within this group of 18, variants that are in linkage disequilibrium with R^2^ > 0.8 are indicated by the same superscript number (^1,2,3,^ or ^4^) in the Variant ID. Following this group of 18, variants identified in this study are listed in alpha-numeric order by Variant ID.
(XLS)
