# Supplementary material for: A Genetic Signature of Spina Bifida Risk from Pathway-Informed Comprehensive Gene-Variant Analysis
Source: PLoS One. 2011 Nov 30;6(11):e28408. doi: 10.1371/journal.pone.0028408 (PMC3227667; doi:10.1371/journal.pone.0028408)
Supplement: Table S1 — The race-ethnic breakdown of the cases and controls in the study population. (DOC) [file pone.0028408.s001.doc]

Table S1. The race-ethnic breakdown of the cases and controls in the study population

|  | Cases | Controls |
| --- | --- | --- |
| Hispanic | 173 | 138 |
| White, non-Hispanic | 44 | 56 |
| Asian | 9 | 18 |
| Black | 9 | 17 |
| Other/Unknown | 6 | 10 |
| **TOTAL** | **241** | **239** |
