# Supplementary material for: A Genetic Signature of Spina Bifida Risk from Pathway-Informed Comprehensive Gene-Variant Analysis
Source: PLoS One. 2011 Nov 30;6(11):e28408. doi: 10.1371/journal.pone.0028408 (PMC3227667; doi:10.1371/journal.pone.0028408)
Supplement: Table S2 — The list of folate-related genes (with NCBI identifiers) whose coding regions were sequenced. (DOC) [file pone.0028408.s002.doc]

Table S2. The list of folate-related genes (with NCBI identifiers) whose coding regions were sequenced.

|  |  |  |  |  |  |
| --- | --- | --- | --- | --- | --- |
| HGNC NAME | ENZYME | NCBI GeneID | NCBI Protein ID | NCBI Nucleotide ID | Coding Exons |
| *AHCY* | adenosylhomocysteinase | 191 | NP_00687 | NM_000687 | 10 |
| *AHCYL1* | adenosylhomocysteinase-like 1 | 10768 | NP_006612 | NM_006621 | 17 |
| *AHCYL2* | adenosylhomocysteinase-like 2 | 23382 | NP_056143 | NM_015328 | 17 |
| *ALDH1L1* | aldehyde dehydrogenase 1, member L1 | 10840 | NP_036322 | NM_012190 | 22 |
| *ALDH1L2* | aldehyde dehydrogenase 1 , member L2 | 160428 | NP_001029345 | NM_001034173 | 23 |
| *AMT* | aminomethyltransferase | 275 | NP_000472 | NM_000481 | 9 |
| *ATIC* | 5-aminoimidazole-4-carboxamide ribonucleotide formyltransferase/IMP cyclohydrolase | 471 | NP_004035 | NM_004044 | 16 |
| *BHMT* | betaine--homocysteine S-methyltransferase | 635 | NP_001704 | NM_001713 | 8 |
| *BHMT2* | betaine--homocysteine S-methyltransferase 2 | 23743 | NP_060084 | NM_017614 | 8 |
| *CBS* | cystathionine-beta-synthase | 875 | NP_000062 | NM_000071 | 15 |
| *CTH* | cystathionase | 1491 | NP_001893 | NM_001902 | 12 |
| *DHFR* | dihydrofolate reductase | 1719 | NP_000782 | NM_000791 | 6 |
| *DMGDH* | dimethylglycine dehydrogenase | 29958 | NP_037523 | NM_013391 | 16 |
| *FOLH1* | folate hydrolase | 2346 | NP_004467 | NM_004476 | 19 |
| *FPGS* | folylpolyglutamate synthase | 2356 | NP_004948 | NM_004957 | 15 |
| *FTCD* | formiminotransferase cyclodeaminase | 10841 | NP_006648 | NM_006657 | 14 |
| *GART* | phosphoribosylglycinamide formyltransferase | 2618 | NP_000810 | NM_000819 | 21 |
| *GGH* | gamma-glutamyl hydrolase | 8836 | NP_003869 | NM_003878 | 9 |
| *MAT1A* | methionine adenosyltransferase I, alpha | 4143 | NP_000420 | NM_000429 | 9 |
| *MAT2A* | methionine adenosyltransferase II, alpha | 4144 | NP_005902 | NM_005911 | 9 |
| *MTFMT* | mitochondrial methionyl-tRNA formyltransferase | 123263 | NP_640335 | NM_139242 | 9 |
| *MTHFD1* | methylenetetrahydrofolate dehydrogenase I | 4522 | NP_005947 | NM_005956 | 27 |
| *MTHFD2* | methylenetetrahydrofolate dehydrogenase II | 10797 | NP_006627 | NM_006636 | 8 |
| *MTHFR* | methylenetetrahydrofolate reductase | 4524 | NP_005948 | NM_005957 | 11 |
| *MTHFS* | 5,10-methenyltetrahydrofolate synthetase | 10588 | NP_006432 | NM_006441 | 3 |
| *MTR* | 5-methyltetrahydrofolate-homocysteine methyltransferase | 4548 | NP_000245 | NM_000254 | 33 |
| *MTRR* | 5-methyltetrahydrofolate-homocysteine methyltransferase reductase | 4552 | NP_002445 | NM_002454 | 14 |
| *SARDH* | sarcosine dehydrogenase | 1757 | NP_009032 | NM_007101 | 20 |
| *SHMT1* | serine hydroxymethyltransferase 1 | 6470 | NP_004160 | NM_004169 | 11 |
| *SHMT2* | serine hydroxymethyltransferase 2 | 6472 | NP_005403 | NM_005412 | 12 |
| *TYMS* | thymidylate synthetase | 7298 | NP_1062 | NM_001071 | 7 |
|  |  |  |  | **TOTAL** | **430** |
