# Supplementary material for: A Genetic Signature of Spina Bifida Risk from Pathway-Informed Comprehensive Gene-Variant Analysis
Source: PLoS One. 2011 Nov 30;6(11):e28408. doi: 10.1371/journal.pone.0028408 (PMC3227667; doi:10.1371/journal.pone.0028408)
Supplement: Table S3 — Pathway designations for thymidylate synthesis, homocysteine levels and purine synthesis. Gene sets inferred to be relevant for a metabolite are indicated by a “+” or “–” sign, which indicates the inferred effect that gene product exerts on metabolite levels and, thus, whether variants in those genes are added to or subtracted from the Metabolic Index Score (see text for details). Those without a sign are not factored into the pathway-level Metabolic Index Score for that metabolite. (DOC) [file pone.0028408.s003.doc]

Table S3. Pathway designations for thymidylate synthesis, homocysteine levels, purine synthesis. Gene sets inferred to be relevant for a metabolite are indicated by a “+” or “–“ sign, which indicates the inferred effect that gene product exerts on metabolite levels and, thus, whether variants in those genes are added to or subtracted from the Metabolic Index Score (similar to the discussion for purine synthesis earlier). Those without a sign are not factored into the pathway-level Metabolic Index Score for that metabolite.

| **Gene** | **Thymidylate Synthesis** | **Homocysteine Levels** | **Purine Synthesis** |
| --- | --- | --- | --- |
| *AHCY* |  | + |  |
| *AHCYL1* |  |  |  |
| *AHCYL2* |  |  |  |
| *ALDH1L1* |  | + | - |
| *ALDH1L2* |  | + | - |
| *AMT* |  | - | + |
| *ATIC* |  | + | + |
| *BHMT* |  | - |  |
| *BHMT2* |  | - |  |
| *CBS* |  | - |  |
| *CTH* |  | - |  |
| *DHFR* | + | - | + |
| *DMGDH* |  | - | + |
| *FOLH1* |  |  |  |
| *FPGS* |  |  |  |
| *FTCD* |  |  |  |
| *GART* |  | + | + |
| *GGH* |  |  |  |
| *MAT1A* | + |  |  |
| *MAT2A* | + |  |  |
| *mtFMT* |  | + | - |
| *MTHFD1* | - | - | + |
| *MTHFD2* |  | - | + |
| *MTHFR* | - | - |  |
| *MTHFS* |  |  |  |
| *MTR* | + | - |  |
| *MTRR* | + | - |  |
| *SARDH* |  | - | + |
| *SHMT1* | + | + | - |
| *SHMT2* | + | - | + |
| *TYMS* | + | + |  |
